# Whole-Transcriptome Analysis of Yak and Cattle Heart Tissues Reveals Regulatory Pathways Associated With High-Altitude Adaptation

**DOI:** 10.3389/fgene.2021.579800

**Published:** 2021-05-21

**Authors:** Hui Wang, Jincheng Zhong, Jikun Wang, Zhixin Chai, Chengfu Zhang, Jinwei Xin, Jiabo Wang, Xin Cai, Zhijuan Wu, Qiumei Ji

**Affiliations:** ^1^Key Laboratory of Qinghai-Tibetan Plateau Animal Genetic Resource Reservation and Utilization, Southwest Minzu University, Chengdu, China; ^2^Key Laboratory of Qinghai-Tibetan Plateau Animal Genetic Resource Reservation and Utilization, Ministry of Education, Southwest Minzu University, Chengdu, China; ^3^State Key Laboratory of Barley and Yak Germplasm Resources and Genetic Improvement, Tibet Academy of Agricultural and Animal Husbandry Science, Lhasa, China

**Keywords:** cattle, yak, whole-transcriptome, co-differentially expressed transcripts, hypoxic adaptation, ceRNA network

## Abstract

**Background:**

The yak (*Bos grunniens*) is an important livestock species that can survive the extremely cold, harsh, and oxygen-poor conditions of the Qinghai-Tibetan Plateau and provide meat, milk, and transportation for the Tibetans living there. However, the regulatory network that drive this hypoxic adaptation remain elusive.

**Results:**

The heart tissues from LeiRoqi (LWQY) yak and their related cattle (*Bos Taurus*) breeds, which are two native cattle breeds located in high altitude (HAC) and low altitude (LAC) regions, respectively, were collected for RNA sequencing. A total of 178 co-differentially expressed protein-coding transcripts (co-DETs) were discovered in each of the LAC-vs-LWQY and LAC-vs-HAC comparison groups, including *NFATC2*, *NFATC1*, *ENPP2*, *ACSL4*, *BAD*, and many other genes whose functions were reported to be associated with the immune-system, endocrine-system, and lipid metabolism. Two and 230 lncRNA transcripts were differentially expressed in the LAC-vs-LWQY and LAC-vs-HAC comparisons’ respectively, but no lncRNA transcripts that were co-differentially expressed. Among the 58 miRNAs that were co-differentially expressed, 18 were up-regulated and 40 were down-regulated. In addition, 640 (501 up-regulated and 139 down-regulated) and 152 (152 up-regulated and one down-regulated) circRNAs showed differential expression in LAC-vs-LWQY and LAC-vs-HAC comparison groups, respectively, and 53 up-regulated co-differentially expressed circRNAs were shared. Multiple co-DETs, which are the targets of miRNAs/lncRNAs, are significantly enriched in high-altitude adaptation related processes, such as, T cell receptor signaling, VEGF signaling, and cAMP signaling. A competing endogenous RNA (ceRNA) network was constructed by integrating the competing relationships among co-differentially expressed mRNAs, miRNAs, lncRNAs and circRNAs. Furthermore, the hypoxic adaptation related ceRNA network was constructed, and the six mRNAs (*MAPKAPK3*, *PXN*, *NFATC2*, *ATP7A*, *DIAPH1*, and *F2R)*, the eight miRNAs (including miR-195), and 15 circRNAs (including novel-circ-017096 and novel-circ-018073) are proposed as novel and promising candidates for regulation of hypoxic adaptation in the heart.

**Conclusion:**

In conclusion, the data recorded in the present study provides new insights into the molecular network of high-altitude adaptation along with more detailed information of protein-coding transcripts and non-coding transcripts involved in this physiological process, the detailed mechanisms behind how these transcripts “crosstalk” with each other during the plateau adaptation are worthy of future research efforts.

## Introduction

The yak (*Bos grunniens*) is an indigenous and rare bovine species distributed across the Qinghai-Tibet Plateau and adjacent areas at altitudes above 2500 m. Archeological evidence indicates that yak and domestic cattle (*Bos taurus*), which both originated from wild origin cattle, are two closely related species which diverged five million years ago (Beja-Pereira et al., 2006; Qiu et al., 2012). Previous studies have shown that the genomes of these two species share strong similarities, as both are composed of 30 chromosomes and similar karyotypes (Wiener et al., 2003), and there is extensive synteny in the two ruminants’ genomes (Qiu et al., 2012). Cattle suffer from sever pulmonary hypertension when translocated to yak inhabited habitats (Hecht et al., 1962; Weir et al., 1974). Through evolution over millions of years, yak have developed many anatomical and physiological traits that enable them to survive at high altitudes, such as larger lungs and hearts (Wiener et al., 2003), a shorter tongue (Shao et al., 2010), stronger environmental sensing (Wiener et al., 2003), higher energy metabolism (Wiener et al., 2003; Wang et al., 2011), and a lack of hypoxic pulmonary vasoconstriction (Wiener et al., 2003). Recent studies on the morphology (Hoppeler et al., 1990), anatomy (Ahmad et al., 2016), physiology (Monge and Leon-Velarde, 1991), genomics (Ji et al., 2020; Wiener et al., 2003), mRNA (Wang et al., 2016; Qi et al., 2019), and small RNAs (Guan et al., 2017) of yak have focused on the high-altitude adaptation mechanisms, but research centered on the whole transcriptome and their “crosstalk” network behind high altitude adaptation is limited.

The term “universal whole transcriptome” refers to all transcripts in tissue or cells, including mRNA and non-coding RNA (ncRNA) (Liu et al., 2018). Among the different kinds of ncRNAs, it appears that microRNA (miRNA), long noncoding RNA (lncRNA), and circular RNA (circRNA) receive the most research attention, the regulatory functions of which are associated with mRNA (Chen and Huang, 2018; He et al., 2018). MiRNA and lncRNA are two classes of endogenous, linear ncRNAs, which play important roles in various biological and pathological processes, including the hypoxia adaptive response (Shih et al., 2017; Choudhry and Harris, 2018), lipid and glucose metabolism (Massart et al., 2017; Goyal et al., 2018), and carcinogenesis and tumor progression (Anastasiadou et al., 2018). However, the regulatory mechanism of these two kinds of ncRNAs are different. MiRNAs generally post-transcriptionally down regulate mRNA stability or block mRNA translation by binding to the 3′-untranslated region (3′-UTR) of specific mRNA targets in plants and animals (Filipowicz et al., 2008). It has been shown that 70 miRNAs are differentially expressed between yak and cattle lung, and those miRNAs are associated with hypoxia-related functions (Guan et al., 2017). lncRNA, as a transcription regulator, can affect gene expression not only through chromatin modification, but also via base pairing of mRNA and using decoys of RNA-binding proteins/miRNAs (Wang and Chang, 2011). Previous studies have shown that the MLL1 histone methylases coordinates with hypoxia-inducible factor 1α(HIF1α) and histone acetyltransferase p300 to regulate hypoxia-induced lncRNA HOTAIR expression (Bhan et al., 2017). In addition, recent studies have shown that circRNA can inhibit miRNA function by acting as miRNA sponges, and can modulate gene expression both transcriptionally and post-transcriptionally (such as mRNA splicing and stability) (Hansen et al., 2013). Furthermore, circRNAs can interact with proteins to regulate gene expression. To explain the correlation between genome size and organism complexity, Salmena et al. raised the “ceRNA hypothesis” which hypothesizes that mRNA, transcribed pseudogenes, and lncRNA “talk” to each other through miRNA response elements (MREs), then form large-scale regulatory networks across the transcriptome (Salmena et al., 2011). Previous studies on the yak intramuscular fat deposition regulation network have revealed that miR-381-y-*TCONS-00016416*-*SIRT1*, miR-122-X-*TCONS-00061798*-*PRKCA* and miR-499-y-*TCONS-00084092*-*LPL* are three potential ceRNA networks with key roles in intramuscular fat deposition (Wang et al., 2020).

In the present study, heart tissues of Leiwoqi yak (LWQY) and two local cattle breeds located at different altitudes were used to construct RNA libraries, and high throughput sequencing was performed to investigate: (I) whether ncRNAs were involved in the mechanism behind high altitude adaptation; (II) potential high altitude adaptation related mRNAs and ncRNAs; (III) which pathways these differentially expressed ncRNAs enriched; (IV) whether these ncRNAs “talk” to each other.

## Methods

### Tissue Samples and RNA Extraction

A total of nine healthy, 4.5-year-old, non-pregnant, and unrelated adult Leiwoqi yak females (LWQY, Leiwoqi country, Changdu city, Tibet province, China, altitude: 4200 m above sea level, geographic coordinates: 96°23′33″E 31°27′3″N) and their two related native cattle breeds located at high altitude (HAC, Changdu city, Tibet province, altitude: 4200m above sea level, geographic coordinates: 96°23′33″E 31°27′3″N) and low altitude (LAC, Wenchuan country, Aba city, Sichuan province, China, altitude: 1200m above sea level, geographic coordinates: 103°35′26″E 31°28′36″N), were randomly selected for heart tissue sampling, with each group represented by three healthy individuals of similar body weight. The LWQY yak and the HAC cattle were reared under similar environmental and feeding conditions in Leiwoqi country, while the LAC cattle were reared under the same feeding conditions as LWQY yak and HAC cattle in Wenchuan country. Briefly, all animals were stunned with a captive bolt pistol to ameliorate the suffering before humane killing between Oct 21st to 24^th^, 2017, following which the exsanguination using a transverse incision of the neck was carried out in the local slaughterhouse in Leiwoqi and Wenchuan country, respectively. Then, approximately 2 g left atrium tissues were collected immediately and washed at least three times in sterile phosphate buffered saline (PBS) to remove the residual blood within 20 min, and stored in liquid nitrogen until RNA isolation. Total RNA was extracted using Trizol reagent (Invitrogen, United States) according to the manufacturer’s instructions. The RNA concentration, purity, and integrity were determined using a NanoDrop 2000 spectrophotometer (Thermo Fisher Scientific).

### Next Generation Sequencing and Data Processing

Next generation sequencing (NGS) was performed by Gene Denovo Biotechnology Co. (Guangzhou, China). The miRNA library was prepared for miRNA sequencing according to the experimental procedures reported previously (Guan et al., 2017), and was sequenced using Illumina HiSeq ^TM^ 4000 following the distributor’s recommended protocol. Raw data were successively filtered by removing the following: (1) low quality reads containing more than one low quality (*Q*-value ≤ 20) base or containing unknown nucleotides (N); (2) reads containing 5′ adaptor, no 3′ adaptor, or no insertion sequence; (3) reads containing poly (A) in small RNA fragments or that were shorter than 18 nt (not including the adaptor); (4) Known classes of bovine RNAs (mRNA, rRNA, tRNA, snRNA, scRNA, snoRNA, and repeats), which were removed through searching against three databases, including GenBank database (Release 209.0), Rfam (Release 14.2), and reference Genome. For cattle, all retained reads were then searched against the miRBase database (Release 22) to identify known existing miRNAs, then the unannotated reads were aligned with the cattle genome (*ARS-UCD1.2*) to identify novel miRNAs. For yak, the retained reads were mapped to the known miRNAs in miRBase 22.0 to obtain known miRNAs, and the unannotated reads were aligned with the yak genome (*BosGru v2.0*) to obtain novel miRNAs.

The RNA (mRNA/lncRNA/circRNA) cDNA libraries were generated using the mRNA-Seq sample preparation kit (Illumina) after ribosomal RNA was removed by the Epicentre Ribo-Zero rRNA kit (Epicentre, United States), and the cDNA was sequenced using Illumina HiSeq ^TM^ 4000 following the distributor’s recommended protocol. Further details on the data processing protocols have been previously reported (Ye et al., 2018). In brief, reads with adaptor contamination, low quality bases, and undetermined bases were removed using Cutadapt (version 2.10). The HISAT2 alignment program was used to map the reads to the yak or cattle genome, respectively. Putative protein-coding RNAs were filtered out using a minimum length and exon number threshold. Transcripts of more than 200 nt and with more than two exons were selected as lncRNA candidates for further analysis. The Coding-Non-Coding-Index (version 2) (Sun et al., 2013), Coding Potential Calculator (CPC^1^) (Kong et al., 2007), and phylogenetic codon substitution frequency (PhyloCSF) (Lin et al., 2011) programs were used to predict the protein-coding potentials of new transcripts using default parameters. The intersection of the results without protein-coding potential yielded the lncRNA transcripts.

The CIRI software package^2^ was used to identify circular RNAs (circRNAs) (Gao et al., 2015). Firstly, CIRI employed BWA to align the clean reads with a reference genome to generate SAM files. Then, CIGAR values and junction reads with paired chiastic clipping (PCC) signals were analyzed and detected using the CIRI software. Finally, the SAM files were scanned by CIRI to detect additional junction reads excluding false positive candidates. Differentially expressed circRNAs were identified using the “EBSeq” package in R.

The expressions of all transcripts were estimated using the StringTie-Ballgown software package (Pertea et al., 2016). Furthermore, the edgeR software package^3^ was used to identify differentially expressed transcripts across samples or groups, and mRNA and lncRNA had false discovery rates (FDR) < 0.05 and |log2(Fold Change) |≥1 in comparison as significant differentially expressed transcripts, and miRNA and circRNA exhibited |log2(Fold Change) |≥1 and *P* < 0.05. It is worth noting that one cattle gene ID corresponds to one or more yak gene IDs, and the co-differentially expressed transcripts were analyzed based on cattle gene ID.

### Association Analysis Between lncRNA and mRNA

Previous studies suggest that an increasing number of lncRNA functions in specific physiological and pathological contexts are associated with mRNA expression levels (Wang and Chang, 2011). Therefore, to reveal the association between the lncRNA and mRNA, the antisense-lncRNA, cis-lncRNA, and trans-lncRNA were predicted as follows: (1) LncRNA is involved in the regulation of many post-transcriptional processes, similarly to small RNAs such as miRNA and snoRNA, which are often associated with complementary pairing of bases. A portion of antisense lncRNA may regulate gene silencing, transcription, and mRNA stability via binding to the sense strand of mRNA. To reveal the interaction between antisense lncRNA and mRNA, RNAplex software was used to predict complementary bases between antisense lncRNA and mRNA. (2) The lncRNAs annotated in the “unknown region” were used for cis regulation analysis. The lncRNAs located within 10 kb upstream or downstream of a gene may overlap with the region of the cis-acting element and participate in mRNA transcriptional regulation, these were annotated as cis-lncRNAs. (3) The principle of trans target gene prediction for lncRNA is that the function of the lncRNA is not related to the location of the coding gene, but to co-expression with the protein coding gene. Therefore, co-expression analysis of lncRNAs and protein-coding genes was used to predict the target genes between samples. LncTar (version 1.0) was used to calculate the free energy between lnRNAs and mRNAs and to predict the targets of lncRNAs (Li et al., 2014).

### MiRNA Target Prediction and Construction of the ceRNA Network

Three software packages (MIREAP, miRanda, and TargetScan) were used to predict the miRNA and circRNA targets. MiRNA sequences and family information were obtained from the TargetScan website^4^.

The ceRNA network was constructed based on the ceRNA theory (Häussinger and Schliess, 2007; Hansen et al., 2013) as follows: (1) Expression correlation between mRNA-miRNA or lncRNA-miRNA was evaluated using Spearman’s rank correlation coefficients (SCC), and pairs with SCC < −0.7 were categorized as negatively co-expressed lncRNA-miRNA or mRNA-miRNA pairs. Both mRNA and lncRNA were miRNA target genes, and all RNAs were differentially expressed. (2) Expressed correlation between lncRNA and mRNA was evaluated using Pearson’s correlation coefficient, and pairs with values >0.9 were deemed co-expressed lncRNA-mRNA pairs, both mRNA and lncRNA in this pair were targeted and co-expressed negatively with a common miRNA. (3) Hypergeometric cumulative distribution function tests were used to test whether the common miRNA sponges between the two genes were significant. As a result, only the gene pairs with a *p*-Value less than 0.05 were selected.

P-value=1-F⁢(x/U,M,N)

For a given each gene pair (A, B), all their regulator miRNAs were denoted as miRNA set C (regulating gene A) and D (regulating gene B). In the formula above, X stands for the number of common miRNAs that regulate both of the genes, U represents the total number of miRNAs identified in this work, M presents the size of miRNA set C and N present the size of miRNA set D.

### Functional Enrichment and Visualization of the ceRNA Network

To annotate the function of DEGs, Gene Ontology (GO) enrichment and Kyoto Encyclopedia of Genes and Genomes (KEGG) pathway analyses were performed using the “GOseq” package in R and the DAVID web server annotation tool (version 6.8), respectively. The GO terms of the DEGs were assessed using Fisher’s exact test. Only those scoring *p* < 0.05 were considered statistically significant and listed.

The lncRNA-miRNA-mRNA network was constructed by assembling all co-expressed competing triplets, which were identified as above, and was visualized using Cytoscape software (version 3.6.0^5^).

### Real-Time Quantitation PCR

cDNA was synthesized using the PrimeScript RT reagent (Takara, Ostu, Japan) and quantitative PCR was performed using the SYBR Premix Ex Taq kit (Takara) on a CFX96 Real-time PCR detection system (Bio-Rad, CA, United States) according to the manufacture’s protocol. The qPCR validation was carried out using three biological replicates and repeated three times(*n* = 9), and the primers are listed in Supplementary Table 1-1. Three endogenous control genes (*Bos taurus GAPDH* and U6 small nuclear RNA genes) were used in this assay, and the products were confirmed by agarose gel electrophoresis and Sanger sequencing. The 2^−ΔΔCt^ method was used to determine the expression level of objective transcripts. The RT-qPCR results are expressed as “mean+standard error of the means,” Student *t*-test was employed to analyze the transcript expression both in LAC-vs-HAC and LAC-vs-LWQY comparison groups, *P* < 0.05 was considered significantly different.

## Results

### Overview of RNA-Sequencing

The aim of the present study was to obtain a global view of the yak and cattle hearts transcriptome (including mRNA, lncRNA, miRNA, and circRNA) and to identify whether these elements are related to high altitude adaptability. Two cDNA libraries (a strand-specific library and a miRNA library) were constructed and sequenced for each tissue sample. A total of 973,288,574 raw reads were obtained from strand-specific libraries, with an average of 108,143,174 per library. After a series of strict criteria, a total of 959,028,938 raw reads were defined as high quality reads for identifying the mRNAs, lncRNAs, and circRNAs (Supplementary Table 1-1). Paired-end sequencing of the miRNA library yielded a total of 145,480,110 raw reads and 132,141,703 clean reads from the nine libraries, with an average of 14,682,411 clean reads per library (Supplementary Table 1-2).

### Expression Analysis and Functional Annotation of Protein-Coding Transcripts

**FIGURE 1:**
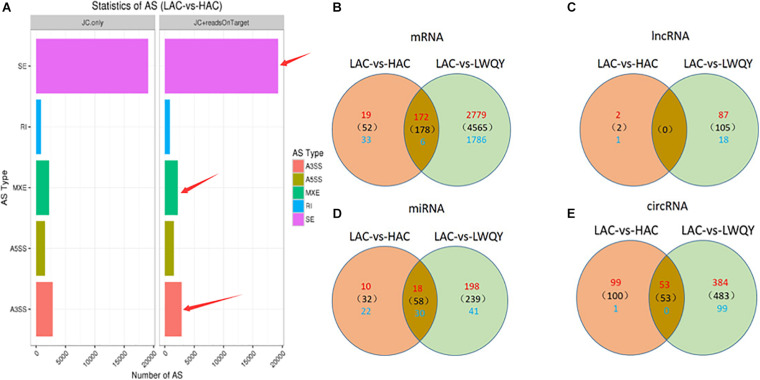
The distribution of alternative splicing events in the HAC and LAC groups **(A)**, and the differentially expressed mRNA transcripts **(B)**, lncRNA transcripts **(C)**, miRNA transcripts **(D)**, and circRNA transcripts **(E)** in the LAC-vs-LWQY and LAC-vs-HAC comparison groups. “SE” is short for “skipped exon,” “A3SS” is short for “alternative 3′ splice site,” “A5SS” is short for “alternative 5′ splice site,” “MXE” is short for “mutually exclusive exon,” “RI” is short for “retained intron,” “HAC” is short for “high-altitude cattle,” “LAC” is short for “low-altitude cattle,” “LWQY” is short for “LeiRoqi yak.”

A total of 63,234 protein-coding transcripts were identified in the cattle strand-specific sequencing libraries, including 44,233 known and 7,957 novel protein-coding transcripts, while 18,891 known and 8,381 novel protein-coding transcripts were identified from yak samples (Table 1 and Supplementary Table 2-1). After transcript abundances were quantified in StringTie-Ballgown, the average expression level of novel protein-coding transcripts (2.93) was found to be about 28% of that of known protein-coding transcripts (10.37) in cattle, and the average expression level of novel protein-coding transcripts (7.43) was about 32% of that of known protein-coding transcripts (23.48) in LWQY (Supplementary Table 2-1). In total, 27,222, 48,767, and 33,235 protein-coding transcripts were detected in the whole LWQY, HAC, and LAC transcripts, respectively (Table 1), and 32,094 yak protein-coding transcripts were mapped to 23,346 cattle transcripts (Supplementary Table 2-1). A total of 18,437 yak transcripts and 14,297 cattle protein-coding transcripts were co-expressed in the LWQY-vs-HAC-vs-LAC comparison groups. A total of 2,351, 1,452, and 185 protein-coding transcripts were uniquely expressed in LWQY, HAC, and LAC, respectively (Supplementary Table 2-1). Furthermore, a large number of alternative splicing events were detected between the LAC and HAC libraries, and 4,742 events were significant between these two groups. The distribution of alternative splicing events in the two groups was similar, with three key type events being prevalent including skipped exons (SE), alternative 3′ splice sites (A3SS), and mutually exclusive exons (MXE) (Figure 1A), implying that the novel protein-coding transcripts identified were mainly generated by these alternative splicing events, which play critical roles in hypoxic adaptation and in the high-altitude diseases of cattle.

**TABLE 1:** Number of transcripts found in the LWQY, HAC, and LAC groups.

Group name	Known isoform Num	New isoform Num	All isoform Num	All reference isoforms
LWQY	18891(65.41%)	8331	27222	28881
HAC	41680(62.74%)	7087	48767	66435
LAC	27791(41.83%)	5444	33235	66435

**FIGURE 2:**
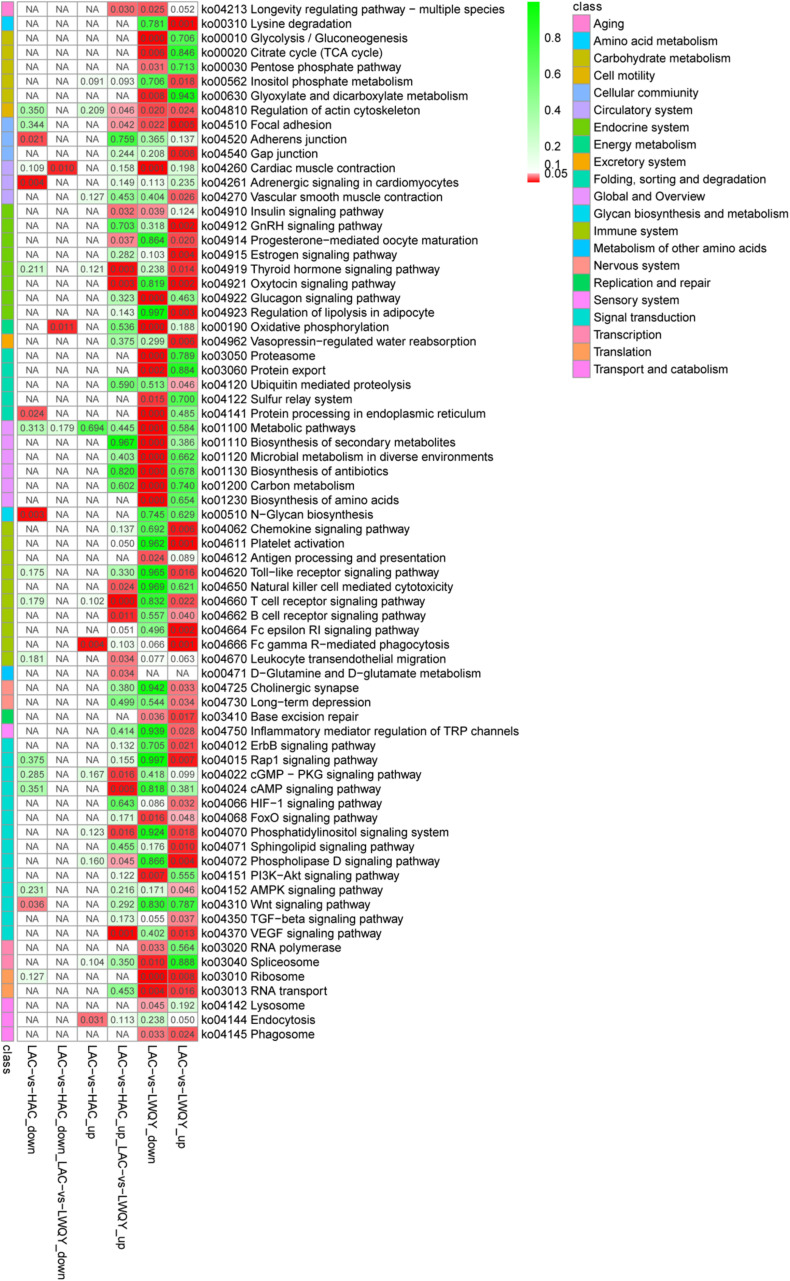
Kyoto Encyclopedia of Genes and Genomes pathway assignments for the 178 differentially co-expressed protein-coding transcripts between the LAC-vs-LWQY and LAC-vs-HAC comparison groups. “KEGG” is short for “Kyoto Encyclopedia of Genes and Genomes.”

Overall, 5,637 (including 3,215 down-regulated and 2,422 up-regulated) and 230 (including 191 down-regulated and 39 up-regulated) significantly differentially expressed transcripts (DETs) were detected in the LAC-vs-LWQY (Supplementary Table 2-2) and LAC-vs-HAC groups (Supplementary Table 2-3), respectively. Additionally, 178 significantly DETs, including six down-regulated and 172 up-regulated, were co-differentially discovered in both the LAC-vs-LWQY and LAC-vs-HAC groups (Figure 1B). These DETs included long-chain-fatty-acid-CoA ligase 4 (ACSL4), tyrosine-protein kinase ZAP-70 (Zap70), T-cell surface glycoprotein CD3 epsilon chain precursor (CD3E), and many other genes that play significant roles in lipid metabolism, immunity regulation, and other physiological processes related to high-altitude adaptation. Gene ontology enrichment (Supplementary Table 2-4) and KEGG pathway analysis (Figure 2 and Supplementary Table 2-5) were carried out to identify the biological functions of the 178 co-differentially DETs (co-DETs) using the cattle reference genome. Among these, the most important cellular component functions involved the cell, cell parts, organelles, and extracellular matrix components. The molecular functions consisted of binding, catalytic activity, nucleic acid binding transcription factor activity, and signal transducer activity. In terms of biology processes, the important GO terms were biological adhesion, immune system processes, response to stimulus, and cell adhesion. In the KEGG pathway analysis, numerous genes were enriched from signal transduction pathways including the immune system and the signal transduction system. It was also discovered that the VEGF (Tong et al., 2006), cAMP (Knerr et al., 2005), oxidative phosphorylation, B/T cell receptor, natural killer cell-mediated cytotoxicity (Vaupel and Multhoff, 2018), and cardiac muscle contraction (Amoasii et al., 2018) signaling pathways were enriched, which are related to hypoxic adaptation systems and are important for oxygen transportation, environmental sensing, energy metabolism, and infection and immune defense systems. There were 15 genes associated with the above mentioned pathways, including cytochrome c oxidase subunit 7C (*COX7C*), mitogen-activated protein kinase-activated protein kinase 3 (*MAPKAPK3*), BCL2 associated agonist of cell death (*BAD*), vav guanine nucleotide exchange factor 1 (VAV1), nuclear factor of activated T cells 2 (*NFATC2*), nuclear factor of activated T cells 1 (*NFATC1*), and phosphoinositide-3-kinase regulatory subunit 5 (*PIK3R5*), and these genes were significantly up-regulated (>10-fold) in the heart tissues of HAC and LWQY compared to LAC. These results indicate that the mechanism behind high-altitude adaptation involves diverse complex cellular processes and changes to related genes and kinases, which are worthy of future research efforts.

The involvement of a few interesting pathways involved in the endocrine system (Supplementary Table 2-5) in the metabolism of other amino acids such as the thyroid hormone signaling pathway (Klecha et al., 2006), D-Glutamine metabolism, and D-glutamate metabolism (Häussinger and Schliess, 2007) were also noted, and previous studies have shown that these pathways were associated with the immune system and protein metabolism in mammals.

### Expression Analysis and Functional Annotation of lncRNA Transcripts

In total, 4,657 lncRNA transcripts were produced, with 1,429, 3,556, and 1,540 lncRNA transcripts detected in the whole transcriptomes of LWQY, HAC, and LAC, respectively (Supplementary Table 3-1). The LWQY lncRNA transcripts corresponded to 1,222 lncRNA genes, while the HAC and LAC lncRNA transcripts corresponded to 2,874 lncRNA genes, and 864 yak lncRNA transcripts (691 lncRNA transcripts genes) were mapped to 689 cattle lncRNA transcripts (620 lncRNA genes). Furthermore, in the LWQY-vs-HAC-vs-LAC comparison groups, 420 lncRNA yak transcripts and 323 cattle lncRNA transcripts were co-expressed, respectively. This difference was caused by the fact that one cattle lncRNA transcript corresponds to one or more yak lncRNA transcripts. In addition, 131, 11, and three lncRNA transcripts were uniquely expressed in LWQY, HAC, and LAC, respectively (Supplementary Table 3-2). The uniquely expressed lncRNAs in the different groups may be a reflection of their physiological role in the regulation of hypoxic adaptation in Bovidae.

**FIGURE 3:**
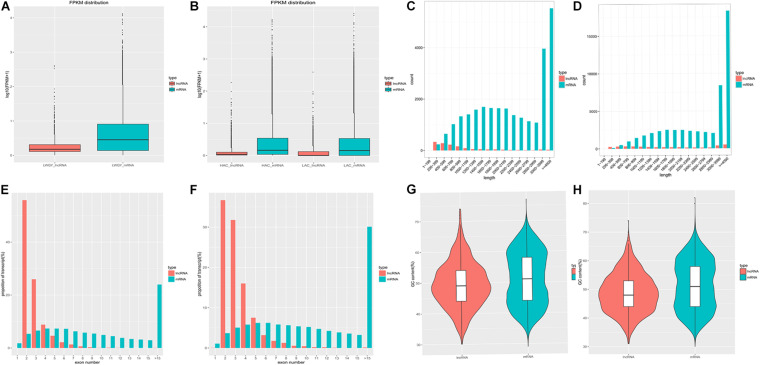
The protein-coding and lncRNA transcripts characteristics in the yak and cattle hearts. **(A,B)** FPKM (Fragment Per Kilobase of transcript per Million mapped reads) for protein-coding and lncRNA transcripts in LWQY **(A)** and cattle **(B)**. **(C,D)** The distribution of read lengths for protein-coding and lncRNA transcripts in LWQY **(C)** and cattle **(D)**. **(E,F)** The proportions of exons per transcript for protein-coding and lncRNA transcripts in LWQY **(E)** and cattle **(F)**. **(G,H)** The GC contents of protein-coding and lncRNA transcripts in LWQY **(G)** and cattle **(H)**.

The average expression levels, lengths, proportions of exons per transcript, and GC contents of the protein-coding and lncRNA transcripts are shown in Figure 3. In the LWQY group, the average expression level of lncRNA transcripts was lower than that of the protein-coding transcripts in all three groups (Figure 3A), the average length of and number of exons in protein-coding transcripts were greater than in lncRNA transcripts (Figures 3C,E), and the number of exons in lncRNAs and protein-coding transcripts were concentrated at two and five exons, respectively (Figure 3E). Similar results were observed in cattle (Figures 3B,D,F). In addition, the average GC contents of the protein-coding and lncRNA transcripts in LWQY were 51.12% and 49.41% (Figure 3G), respectively, while in cattle the average GC contents were 48.79% and 51.38%, respectively (Figure 3D). The present results are in accordance with those of previous studies (Xu et al., 2016; Han et al., 2017; Lan et al., 2017).

In total, two and 230 lncRNA transcripts were differentially expressed (DELs) in the LAC-vs-LWQY and LAC-vs-HAC groups (Figure 2), respectively, but no lncRNA transcripts co-differentially expressed in those two comparison groups with the limited differentially expressed lncRNAs in LAC-vs-LWQY comparison group (Figure 1C). Through *cis*-, *trans*-, and antisense-regulatory relationship analysis, 168 potential target protein-coding genes of the differential lncRNA transcripts were detected. Functional analysis showed that these target genes were significantly enriched in 388 GO terms (304 under biological processes, 32 under cellular components, and 52 under molecular functions), and many terms were related to morphogenesis, immune responses, and gene expression (Supplementary Figure 1). For example, the biological processes included cell morphogenesis, and cell morphogenesis is involved in differentiation, immune system development, regulation of cell differentiation, methylation, and histone deacetylation. In addition, the target genes were enriched in 17 pathways, including PI3K-Akt signaling, vascular smooth muscle contraction, regulation of lipolysis in adipocyte, and platelet activation, which are all associated with environmental adaptation, immune responses, and carbohydrate metabolism pathways and are very important in hypoxia adaptation (Supplementary Figure 2). These results indicate that the differentially expressed lncRNAs took part in several biological processes in the heart during hypoxia adaptation.

### Expression Analysis and Function Annotation of miRNA Transcripts

A total of 1,937 miRNAs were identified in the nine small RNA libraries (Supplementary Table 4-1), which ranged from 18 to 24 nucleotides in length. Altogether, 831 and 1,713 miRNAs were detected in LWQY and cattle, respectively (Supplementary Table 4-1). The top 10 mature miRNAs with the highest expression levels comprised more than 61% of all miRNAs in LWQY, HAC, and LAC, showing a relatively abundant distribution. Furthermore, miR-99-x were the most generally abundant miRNA overall, together accounting for 20%, 8%, and 4% of the total miRNAs in LWQY, HAC, and LAC, respectively (Table 2). A total of 299 and 90 miRNAs were differentially expressed in the LAC-vs-LWQY (Supplementary Table 4-2) and LAC-vs-HAC (Supplementary Table 4-3) comparison groups, respectively. Fifty-eight miRNAs shared co-differential expression in these two comparison groups, including 18 that were up-regulated and 40 that were down-regulated (Figure 1D).

**TABLE 2:** The 10 miRNAs with the highest expression levels among the three groups.

Group	GeneID_bta	LWQY_TPM	HAC_TPM	LAC_TPM
HAC	bta-miR-99a-5p	0	140992	90476.8
	bta-miR-27b	0	92521	103573
	bta-miR-100	0	87496.4	63698.9
	bta-miR-143	0	79705.9	79847.3
	miR-99-x	205927	66295.3	37992
	bta-miR-26a	0	47381.3	51545.2
	bta-miR-125b	0	24096.4	24479.9
	bta-miR-99b	0	19435.1	14934.9
	bta-miR-486	0	17211.3	21674.8
	bta-miR-191	0	16441.1	19773.3
LAC	bta-miR-27b	0	92521	103573
	bta-miR-99a-5p	0	140992	90476.8
	bta-miR-143	0	79705.9	79847.3
	bta-miR-100	0	87496.4	63698.9
	bta-miR-26a	0	47381.3	51545.2
	miR-99-x	205927	66295.3	37992
	bta-miR-1	0	13645.7	25870.9
	bta-miR-125b	0	24096.4	24479.9
	bta-miR-30e-5p	0	11312.7	22686.1
	bta-miR-486	0	17211.3	21674.8
LWQY	miR-99-x	205927	66295.3	37992
	miR-100-x	126293	6829.27	2557.4
	miR-143-y	118596	2960.73	2227.74
	miR-27-y	104840	1448.52	1348.91
	miR-26-x	60165.4	486.511	204.25
	miR-125-x	53805.2	3014.84	1827.79
	miR-486-x	45976.1	2669	1289.46
	let-7-x	33009.7	699.634	484.538
	miR-30-x	30791.9	386.258	199.14
	miR-191-x	20174.3	147.711	113.028

**FIGURE 4:**
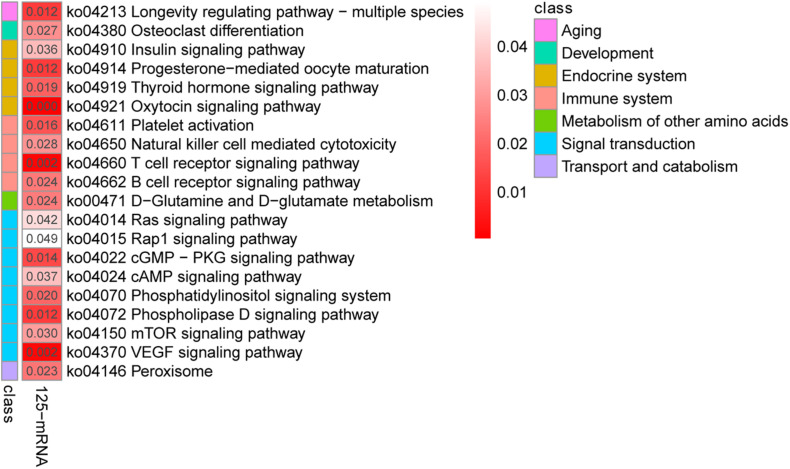
Kyoto Encyclopedia of Genes and Genomes pathway analysis of the 125 differentially co-expressed genes targeted by the 58 differentially co-expressed miRNAs.

To further investigate the functions of these co-differentially expressed miRNAs (co-DEMs), their target genes were predicted and functional annotation was performed. A total of 43,930 target protein-coding genes of the co-DEMs were identified (Supplementary Table 4-4). Among these, 125 genes had transcripts identified as co-DETs and were also significantly negatively correlated with miRNA expression (Supplementary Table 4-5). Thus, they were assigned as intersection genes, and are more likely to be predicted as miRNA target genes in yak and cattle hearts. Similar to the mRNA expression results, the largest numbers of target genes were clustered in biological processes, followed by molecular functions and cellular components (Supplementary Table 4-6). Further, KEGG analysis revealed that these intersection genes were involved T cell receptor signaling, VEGF signaling, and cAMP signaling (Figure 4). Together, these results indicate that miRNAs regulate a variety of processes to adapt yaks to hypoxic, cold, and severe environments.

### CircRNAs Expression Analysis and circRNA-miRNA-Pathway Relationship Network Construction

A total of 23,014 circRNAs were obtained across the nine libraries using the CIRI software. Of these, 15,283 (66.38%) were generated from the exons of protein-coding genes, 1,102 (4.79%) were from intergenic regions, 1,813 (7.87%) were from exon-intron, and 1,739 (7.55%) were from introns. The remainder (3,077, 13.36%) were antisense, and the average length of cattle circRNAs was 2,404 bp. On the other hand, a total of 19,540 circRNAs were obtained from the yak libraries, of which 12,293 (62.97%) were generated from exons of protein-coding genes, 1,610 (8.25%) from intergenic regions, 2,308 (11.82%) from exon-intron, and 854 (4.37%) from introns. The remainder (2,475, 12.68%) were antisense, and the average length of yak circRNAs was 2,719 bp (Supplementary Table 5-1). In addition, 19,522, 25,943, and 11,137 circRNAs were obtained from the LWQY, HAC and LAC groups, respectively (Supplementary Table 5-1). These results suggest that the circRNAs in yak and cattle hearts are from diverse genomic regions, and that the average circRNA length differs among species.

A total of 640 (501 up-regulated and 139 down-regulated) and 152 (152 up-regulated and one down-regulated) circRNAs showed differential expression in the LAC-vs-LWQY (Supplementary Table 5-2) and LAC-vs-HAC (Supplementary Table 5-3) comparison groups, respectively, and 53 up-regulated co-differentially expressed circRNAs (co-DECs) were shared (Figure 1E). It has been reported that circRNAs can bind to miRNAs and consequently repress their function. To determine whether the 53 co-DECs could affect gene post-transcriptional regulation by binding to miRNAs, three different software packages were used to predict their target miRNAs. Interesting, these co-DECs could bind to 881 miRNAs, and 57 of these miRNAs had transcripts identified as being co-differentially expressed and also significantly negatively correlated with 125 co-DETs (Supplementary Table 5-4). This suggests that cirRNAs play important roles as miRNA sponges across a wide array of biological processes in the heart to protect yak from the harsh environment of the plateau.

### Construction of the ceRNA Network

**FIGURE 5:**
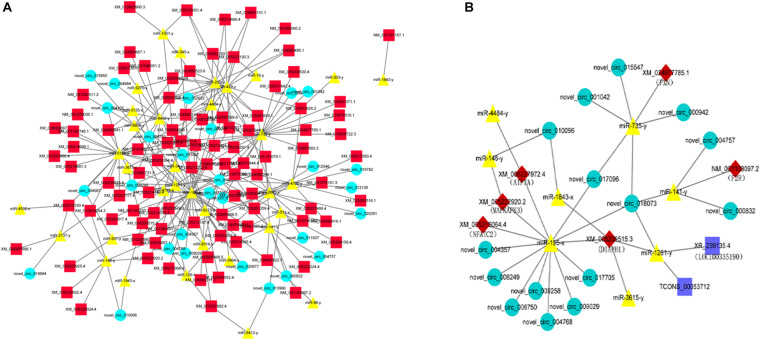
An overview of the competing endogenous RNA (ceRNA) network **(A)** and the predicted hypoxic ceRNA network **(B)**. The red squares, green circles, and yellow triangles indicate co-DETs, co-DEMs, and co-DECs, respectively. The dark blue rectangle indicates the differentially expressed lncRNA transcripts in the LAC-vs-LWQY comparison group.

Based on the co-DETs, co-DECs, and co-DEMs in the LAC-vs-LWQY and LAC-vs-HAC groups, three software packages (MIREAP, miRanda, and TargetScan) were used to identify the biological targets of each miRNA from the protein-coding and circRNA transcripts that showed a significantly negative correlation with miRNA expression. This allowed the mRNA-miRNA and circRNA-miRNA pairs to be identified, and the mRNA-miRNA pairs in which the mRNA was differentially expressed in the two comparison groups were retained. Then, the competing endogenous RNA (ceRNA) network was constructed, which revealed up-regulated miRNAs with decreased expression of protein-coding and circRNA transcripts, while the down-regulated miRNAs showed the reverse results. There were 190 nodes (Figure 5A), which suggested that the ceRNA network was very dense.

Based on the ceRNA network, eight miRNAs and 15 circRNAs were predicted to directly or indirectly interact with six mRNAs (*MAPKAPK3* (Mann et al., 2019), *PXN* (Veith et al., 2016), *NFATC2* (Peng et al., 2001), *ATP7A* (Xie and Collins, 2011), *DIAPH1*, and *F2R*), which were reported to be involved in hypoxia adaptation-related signaling pathways. Subsequently, a hypoxic sub-ceRNA network was constructed (Figure 5B). It is worth mentioning that the two lncRNAs (LOC100335190 and TCONS_00053712) were the lncRNAs that were differentially expressed in the LAC-vs-LWQY comparison group. In the hypoxic ceRNA network, nine novel circRNA transcripts were predicted to interact with *MAPKAPK3*, *NFATC2*, *ATP7A*, and *DIAPH1* through miR-195-x, which indicates that miR-195-x may play an important role in yak hypoxic adaptations in the plateau.

### Validation of the RNA Sequencing Data

**FIGURE 6:**
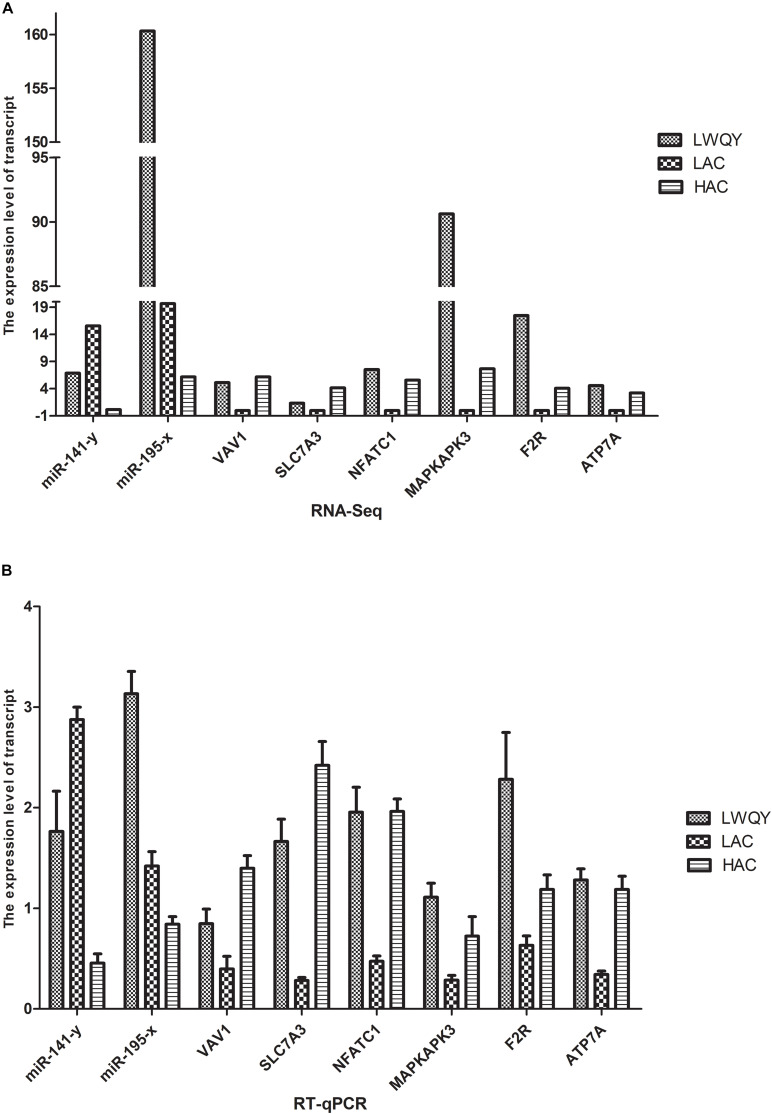
Validation of differentially expressed genes. **(A)** The RNA-seq results of the transcripts. **(B)** the RT-qPCR results of the transcripts.

Validation of the high throughput sequencing results was performed with the quantitative reverse-transcription polymerase chain reaction (RT-qPCR) for three co-DETs (*MAPKAPK3*, *F2R*, and *ATP7A*) and two co-DEMs (miR-195-x and miR-141-y) in the hypoxic adaptation ceRNA network. Expression of the selected transcripts was significantly different both in LAC-vs-HAC and LAC-vs-LWQY groups, which may be closely related to hypoxic adaptation in the ceRNA network (Figure 6). Similarly, three genes were randomly selected from the co-DETs, including *VAV1*, solute carrier family 7 member 3(*SLC7A3*), and *NFATC1*. The data indicated that the expression patterns were highly consistent between the two methods. For example, according to the high throughput sequencing, SLC7A3 was upregulated both in LWQY and HAC compared with LAC group, and the expression patterns were highly consistent with that of the RT-qPCR results (Figure 6). The RT-qPCR results confirmed the high reproducibility and reliability of the gene expression profiling in our study.

## Discussion

High-altitude adaptation is an extremely complex biological process that involves millions of molecules and biological pathways. A large number of SNPs and copy number variations (CNVs) have been researched, and has revealed many genes that may be targets for further high-altitude adaptation mechanism studies in pigs (Tang et al., 2017) and human (Yang et al., 2017). Accumulating evidence has pointed to the fact that small non-coding RNAs, long non-coding RNAs, and circular RNAs are associated with hypoxia signaling pathways in different cell types. Yak provide an ideal model for deciphering the mechanisms behind high-altitude adaptation as they are well-adapted to harsh and extreme environments. Despite the identification of SNPs, CNVs (Wang et al., 2019a), mRNA, and miRNA (Qi et al., 2019) expression profiles for yak adaptations, the whole transcriptome and the connection between mRNA and non-coding RNAs remains poorly understood. Comparing the whole transcriptomes of yak and cattle can provide insight into the regulatory mechanisms behind high-altitude adaptation for a comprehensive understanding, including among mRNAs, miRNAs, lncRNAs, and circRNAs. In the current study, whole transcriptomes analyses of the heart of Leiwoqi (LWQY) yak and their related cattle breeds located in the LWQY yak habitat (HAC) and in a low-altitude region (LAC) were performed, and comparable numbers of transcriptome species were identified using the yak and cattle genomes. In addition, a hypoxia-adaptation ceRNA network was constructed.

Kyoto Encyclopedia of Genes and Genomes analysis revealed that the DETs were enriched in hypoxia-adaptation related pathways, in which some important oxygen transportation-, environmental sense-, energy metabolism-, and immune-relevant genes, such as *COX7C*, *MAPKAPK3*, *PIK3R5*, *NFATC1*, and *NFATC1* were significantly up-regulated in HAC and LWQY compared to in LAC. COX7C is one of the subunits of cytochrome c oxidase (COX), which is the terminal component of the mitochondrial respiratory chain. As a regulator of hydrogen ion transmembrane transport, COX7C plays an important role in energy metabolism. MAPKAPK3 is a serine/threonine protein kinase of the p38 signaling pathway that is activated by a variety of stress stimuli and has been implicated in cellular responses and gene regulation during adaptation to hypoxia. PIK3R5 is a subunit of phosphoinositide 3-kinase γ, which is a lipid kinase that belongs to the IB class subdivision of the Phosphoinositide 3-kinases (PI3Ks). Previous studies have shown that the activated PI3K-Akt pathway in hypoxic endothelial cells is essential for cell survival and angiogenesis, and that it is involved in the hypoxia-inducible factor 1 α signaling pathway (Zhou et al., 2004; Hung et al., 2007). Hence, it is possible that the activated PIK3R5 in the yak heart may play a key role in protecting cells from hypoxia via the PI3K-AKT signaling pathway. NFAT transcription factors play critical roles in gene transcription during immune responses, as the two most prominent NFAT family members, NFATC1 and NFATC2 play an important role in controlling T and B cell activation and differentiation (Macian, 2005; Giampaolo et al., 2019). In addition, the results of the present study showed that the DETs were not only associated with the above-mentioned pathways but also with other pathways involved in hypoxic adaptation, for example the VEGF signaling pathway and the cardiac muscle contraction pathway. Taken together, these results indicate the potential roles of these DETs in high altitude adaptation through the activation of important pathways.

lncRNA is a major class of ncRNAs that may act as important regulator of gene expression in a wide variety of biological processes, including energy balance and immune responses. It has been reported that energy stress-induced lincRNA-FILNC1 could represses c-Myc-mediated energy metabolism and that lincRNA-Cox2 could be induced in mouse macrophages after activation of Toll-like receptors so as to detect microbes and alter the immune system to respond (Xiao et al., 2017). The present experiment is the first to show the expression profiles of lncRNA transcripts in yak hearts and to identify differentially expressed lncRNA transcripts in response to high-altitude using whole transcriptome sequencing. Through functional analysis, 168 target genes of differentially expressed lncRNA transcripts were enriched in 388 GO terms and 17 signaling pathways, which were related to the immune response, carbohydrate metabolism, and environmental adaptation. These results demonstrate that lncRNAs may play important roles in the adaptability of animals to survive on the plateau. In contrast to the protein-coding transcripts, no co-DELs were identified between the LAC-vs-LWQY and LAC-vs-HAC groups, although a substantial number of differentially expressed lncRNAs that may be associated with high altitude adaptation were identified. Such discrepancy in these results may be partially explained by the fact that lncRNAs are often less conserved at the primary sequence level, and this hinders the discovery of lncRNA orthologs in yak and cattle genomes using sequence homology, as since diverging 5 million years ago, yak and cattle have undergone distinct selective pressures resulting in inherent genetic difference which may disturb the results to some extent. Although whole transcriptome data of the yak and its relatives did not reveal co-DELs, the link between the differentially expressed lncRNAs in the LAC-vs-LWQY and LAC-vs-HAC groups and hypoxia adaptation did appear to be biologically relevant.

In this study, a total of 58 co-DEMs were identified between the LAC-vs-LWQY and LAC-vs-HAC groups, which is a greater number than that reported by a previous study (29 miRNAs) (Qi et al., 2019) comparing the miRNA expression profiles in the heart tissues of yak and cattle. The aforementioned previous study concluded that the target genes of the heart miRNAs were mainly associated with hypoxia-related functions (Guan et al., 2017), including the HIFa, PI3K-Akt, and p53 signaling pathways. However, the present study found that the co-differentially expressed target genes of the co-differentially expressed miRNAs were significantly involved in the VEGF, cAMP, T cell receptor, and B cell receptor signaling pathways, which are involved in energy metabolism, immunity, and angiogenesis. Interestingly, the targets were also involved in the longevity-regulating pathway, osteoclast differentiation, and more. Hence, it is likely that the co-differentially expressed miRNAs may be involved in the regulation of not only immune-, energy-, and angiogenesis responses but also of other biology processes.

In addition to mRNAs, lncRNAs, and miRNAs, circRNAs are a distinct class of newly discovered endogenous ncRNAs. Indeed, circRNAs have been found to play crucial roles in various biological and pathological processes across a wide range of organisms (Dai et al., 2018; Xu et al., 2018). However, little is known about the functions of circRNAs in the yak. To explore the expression profiles of circRNAs and their potential functions in regulating high-altitude adaptation, the circRNAs that were differentially expressed between the yak and its relatives were examined. This led to a total of 53 circRNAs showing co-differential expression between the LAC-vs-LWQY and LAC-vs-HAC comparison groups. An analysis of circRNA-miRNA associations showed that 53 co-DECs regulated 57 co-DEMs, suggesting that circRNAs may also play vital roles in regulating a wide range of signaling pathways, hence their involvement in the regulatory mechanisms behind high-altitude adaptation.

The integration of whole transcriptome can generate new information and improve classification accuracy above and beyond analysis of single datasets alone (Zhang et al., 2010; Vilne and Schunkert, 2018). For instance, Liu et al. (2018) demonstrated that the integration of whole transcriptome improved the validity of the functional analysis of differentially expressed miRNAs using intersection genes, and constructed a ceRNA relationship network in broody chickens. Drake et al. (2016) integrated genomic, transcriptomic, and phosphoproteomic datasets to reveal a map of activated signaling pathways in castration-resistant prostate cancer. Xin et al. (2020) compared the proteomic profiles of gluteus between yak and cattle to reveal the molecular mechanisms underlying yak adaptation, which shows that the number of differentially expressed proteins in Tibetan cattle (one cattle breed located in Qinhai-Tibet plateau) vs. yak comparison group is significantly lower than that in Sanjiang cattle (one cattle breed located in Wenchuan contry, Sichuan province) vs. yak comparison group (Xin et al., 2020), we speculate that, to some extent, the mechanism in hypoxic adaptation between yak and HAC is different. Although we found substantial differentially expressed transcripts and constructed the ceRNA network that may be associated with high altitude adaptation, the limitation is that the difference between LWQY and HAC were not compared. A more comprehensive association study of the transcriptome and proteomic is necessary in order to better reveal the mechanism of hypoxic adaptation in yak.

## Conclusion

In summary, this study has investigated the whole transcriptome profiles of nine hearts from the yak and its relatives (cattle in yak habitat and low-altitude cattle). A total of 178 protein-coding transcripts, 58 miRNAs, and 53 circRNAs were identified as being differentially co-expressed between the LAC-vs-LWQY and LAC-vs-HAC comparison groups, and *in silico* analysis indicated that those transcripts can regulate a wide range of biological processes involved in high-altitude adaptation. More interestingly, we obtained eight miRNAs (including miR-195-x) and 15 circRNAs directly or indirectly interacted with six protein-coding transcripts (*MAPKAPK3*, PXN, *NFATC2*, *ATP7A*, *DIAPH1*, and *F2R*) in the hypoxic adaptation ceRNA network. This data provides promising candidate transcripts and “crosstalk” network for elucidating molecular mechanisms controlling high-altitude adaptation in yak and should be explored further.

## Data Availability Statement

The raw RNA sequencing data generated during the current study has been uploaded in the NCBI BioProject with the accession number PRJNA644042.

## Ethics Statement

The animal study was reviewed and approved by The Administration of Affairs Concerning Experimental Animals (Ministry of Science and Technology, China, revised in June 2004) and the Institution Animal Care and Use Committee in the Southwest Minzu University, Chengdu, China. Written informed consent for participation was not obtained from the owners because we obtained the oral informed consent from the owners for the participation of the animals in this study.

## Author Contributions

QJ and JZ were responsible for the experimental design. HW, JKW, and CZ collected the tissue samples and isolated RNA for sequencing. JBW, XC, ZW, JX, and CZ performed the analysis of the sequencing data and bioinformatics analysis. HW wrote the manuscript. HW, QJ, and ZJ supervised the entire experiment and participated in manuscript revision. All authors read and approved the final manuscript.

## Conflict of Interest

The authors declare that the research was conducted in the absence of any commercial or financial relationships that could be construed as a potential conflict of interest.

## Footnotes

1 http://cpc2.cbi.pku.edu.cn/

2 https://sourceforge.net/projects/ciri/

3 http://www.bioconductor.org/packages/release/bioc/html/edgeR.html

4 http://www.targetscan.org/

5 http://www.cytoscape.org/

## Supplementary Material

Supplementary Figure 1 Gene ontology enrichment analysis for the target genes of the differentially expressed lncRNA transcripts.

Supplementary Figure 2 Kyoto Encyclopedia of Genes and Genomes pathway analysis of the target genes of the differentially expressed lncRNA transcripts.

Supplementary Table 1 The primer information for RT-qPCR (Supplementary Table 1-1), and overview of strand-specific (Supplementary Table 1-2) and small RNA sequencing (Supplementary Table 1-3) data.

Supplementary Table 2 Overview of known and novel protein-coding transcripts in cattle and the yak (Supplementary Table 2-1); the differentially expressed mRNAs in LAC-vs-LWQY (Supplementary Table 2-2) and LAC-vs-HAC (Supplementary Table 2-3) comparison groups, respectively; and gene ontology enrichment (Supplementary Table 2-4) and KEGG pathway (Supplementary Table 2-5) analysis of the co-differentially expressed transcripts.

Supplementary Table 3 Overview of lncRNA (Supplementary Table 3-1) and the lncRNA transcripts that were uniquely expressed in the different groups (Supplementary Table 3-2).

Supplementary Table 4 Overview of miRNAs found for the different groups (Supplementary Table 4-1); the differentially expressed miRNAs in the LAC-vs-LWQY (Supplementary Table 4-2) and LAC-vs-HAC (Supplementary Table 4-3) comparisons. Detailed target information for the co-differentially expressed miRNAs (Supplementary Table 4-4), and detailed information on the 125 identified co-DETs, which were also the targets of the co-DEMs (Supplementary Table 4-5), and their GO analysis results (Supplementary Table 4-6).

Supplementary Table 5 The overview of the circRNAs found for the different groups (Supplementary Table 5-1), and the differentially expressed miRNAs in the LAC-vs-LWQY (Supplementary Table 5-2) and LAC-vs-HAC (Supplementary Table 5-3) comparisons, respectively. List of differentially co-expressed miRNAs and protein-coding transcripts linked to 53 differentially co-expressed circRNAs in the LAC-vs-LWQY and LAC-vs-HAC comparison groups (Supplementary Table 5-4).
