# Supplementary figures and images for: Whole-Transcriptome Analysis of Yak and Cattle Heart Tissues Reveals Regulatory Pathways Associated With High-Altitude Adaptation
Source: Front Genet. 2021 May 21;12:579800. doi: 10.3389/fgene.2021.579800 (PMC8176224; doi:10.3389/fgene.2021.579800)

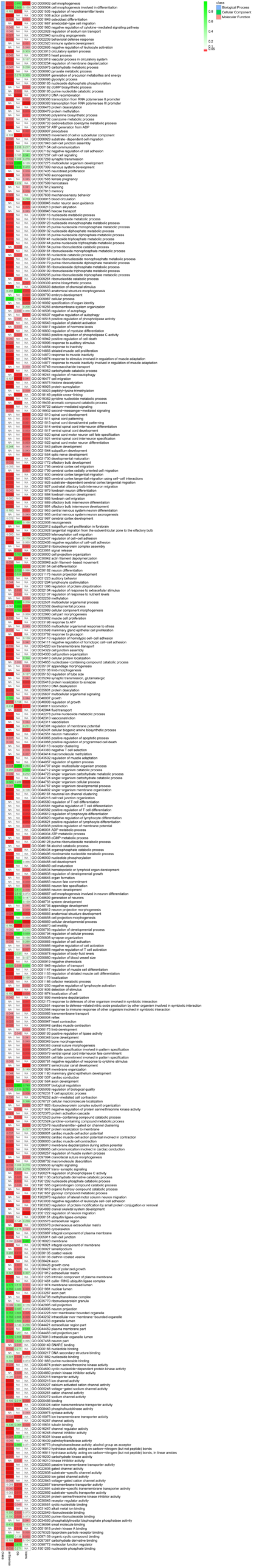

Supplement: Supplementary Figure 1 — Gene ontology enrichment analysis for the target genes of the differentially expressed lncRNA transcripts. [file Image_1.TIF]

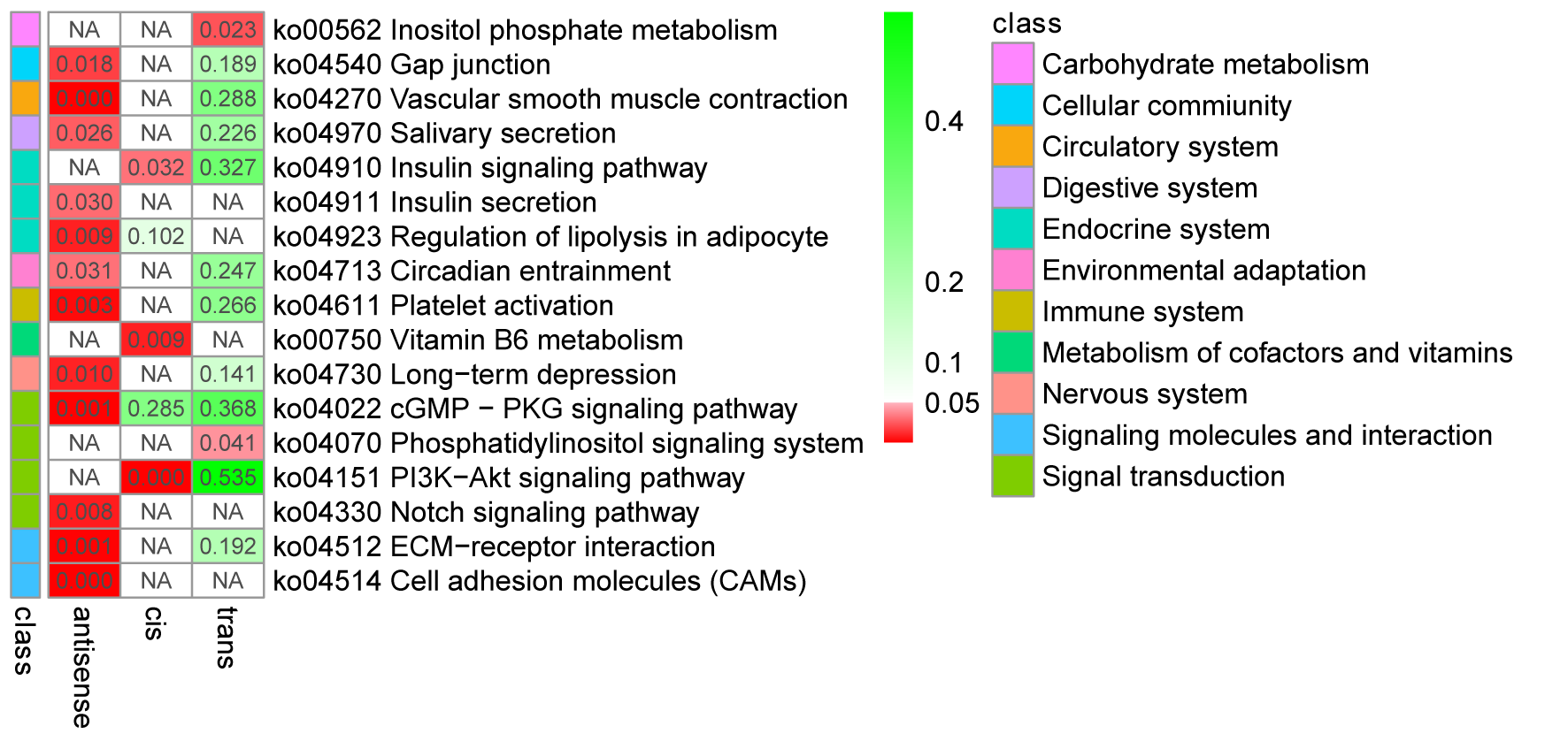

Supplement: Supplementary Figure 2 — Kyoto Encyclopedia of Genes and Genomes pathway analysis of the target genes of the differentially expressed lncRNA transcripts. [file Image_2.TIF]
